# Associations between long-term lithium treatment and renal, thyroid, and parathyroid function: A register-based study

**DOI:** 10.1192/j.eurpsy.2023.1073

**Published:** 2023-07-19

**Authors:** A. C. Wiuff, B. D. Jensen, A. A. Nierenberg, O. Köhler-Forsberg

**Affiliations:** 1 Department of Affective Disorders, Aarhus University Hospital - Psychiatry; 2 Department of Clinical Medicine, Aarhus University, Aarhus, Denmark; 3 Dauten Family Center for Bipolar Treatment Innovation, Massachusetts General Hospital; 4 Harvard Medical School, Boston, United States; 5 Psychosis Research Unit, Aarhus University Hospital - Psychiatry, Aarhus, Denmark

## Abstract

**Introduction:**

Although the effect of lithium treatment on kidney and endocrine systems has been extensively investigated, this literature, however, suffers from substantial heterogeneity and many prior studies are limited by short follow-up on just one marker of interest.

**Objectives:**

We aimed to determine the impact of long-term lithium therapy on renal, thyroid and parathyroid function within a large real-world cohort.

**Methods:**

We performed a cohort study within the Central Region of Denmark (approximately 1.3 million inhabitants). Using the Electronic Patient Record system, we identified all patients with at least one serum-lithium (se-Li) measurement in the period from January 1, 2013 to July 20, 2022, and a reference group of patients diagnosed with bipolar disorder (ICD-10: F30, F31) was matched on age, sex and creatinine level. The outcomes were renal, thyroid, and parathyroid function as indicated by all blood tests taken during follow-up measuring creatinine, estimated glomerular filtration rate (eGFR), thyroid-stimulating hormone (TSH), parathyroid hormone (PTH) and calcium. Multilevel regression analyses adjusted for age, sex, severity of the mental disorder (as indicated by the number of hospitalizations), and somatic comorbidity calculated the association between lithium treatment and development in renal, thyroid, and parathyroid function over time.

**Results:**

A total of 4,709 lithium users (61.5% females, median age 46 years [IQR: 32-60]) and 4,027 control individuals were identified with a total follow-up period of 14,686 person-years (median = 1.7 years, range: 1-9.5). Out of the 4,709 lithium users, a total of 3,157 were incident lithium users. The final results will be shown at the 2023 EPA Congress.

**Conclusions:**

The conclusions will be presented at the congress.

**Disclosure of Interest:**

None Declared

